# New Books

**Published:** 2007-06

Climate Change: What It Means for Us, Our Children, and Our Grandchildren

Joseph F.C. DiMento, Pamela M. Doughman, eds.

Cambridge, MA:MIT Press, 2007. 232 pp. ISBN: 0-262-04241-X, $60

Congenital Diseases and the Environment

P. Nicolopoulou-Stamati, L. Hens, C.V. Howard, eds.

New York:Springer, 2007. 471 pp. ISBN: 1-4020-4830-2, $185

Darwin’s Gift: to Science and Religion

Francisco Ayala

Washington, DC:National Academies Press, 2007. 256 pp. ISBN: 0-309-10231-6, $24.95

Food Toxicants Analysis: Techniques, Strategies and Developments

Yolanda Picò, ed.

Burlington, MA:Elsevier, 2007. 786 pp. ISBN: 0-444-52843-1, $195

Fueling our Future: An Introduction to Sustainable Energy

Robert L. Evans

Cambridge, UK:Cambridge University Press, 2007. 192 pp. ISBN: 0-521-86563-0, $109.99

Globalisation and Sustainable Development

Vladimir F. Krapivin, Costas A. Varotsos

New York:Springer, 2007. 308 pp. ISBN: 3-540-70661-8, $129

Handbook on the Toxicology of Metals, 3rd ed.

Gunnar Nordberg, Bruce Fowler, Monica Nordberg, Lars Friberg, eds.

Burlington, MA:Elsevier, 2007. 1,024 pp. ISBN: 0-12-369413-2, $299.95

Histopathology of Preclinical Toxicity Studies, 3rd ed.

Peter Greaves

Burlington, MA:Elsevier, 2007. 960 pp. ISBN: 0-444-52771-0, $159

Peace Parks: Conservation and Conflict Resolution

Saleem H. Ali, ed.

Cambridge, MA:MIT Press, 2007. 432 pp. ISBN: 0-262-01235-9, $72

Phytoremediation: Methods and Reviews

Neil Willey, ed.

New York:Springer, 2007. 516 pp. ISBN: 1-58829-541-5, $135

Plutonium: A History of the World’s Most Dangerous Element

Jeremy Bernstein

Washington, DC:National Academies Press, 2007. 208 pp. ISBN: 0-309-10296-0, $27.95

Quantitative Structure-Activity Relationships (QSAR) for Pesticide Regulatory Purposes

Emilio Benfenati, ed.

Burlington, MA:Elsevier, 2007. 532 pp. ISBN: 0-444-52710-9, $210

Resisting Global Toxics: Transnational Movements for Environmental Justice

David Naguib Pellow

Cambridge, MA:MIT Press, 2007. 336 pp. ISBN: 0-262-16244-X, $62

Rivertown: Rethinking Urban Rivers

Paul Stanton Kibel, ed.

Cambridge, MA:MIT Press, 2007. 232 pp. ISBN: 0-262-11307-4, $55

The Next Catastrophe: Reducing Our Vulnerabilities to Natural, Industrial, and Terrorist Disasters

Charles Perrow

Princeton, NJ:Princeton University Press, 2007. 388 pp. ISBN: 0-691-12997-6, $29.95

The Working Landscape: Founding, Preservation, and the Politics of Place

Peter F. Cannavò

Cambridge, MA:MIT Press, 2007. 416 pp. ISBN: 0-262-03364-X, $70

Understanding Multiple Environmental Stresses: Report of a Workshop

Committee on Earth–Atmosphere Interactions, National Research Council

Washington, DC:National Academies Press, 2007. 154 pp. ISBN: 0-309-10331-2, $33.75

Validation of Toxicogenomic Technologies: A Workshop Summary

Committee on Validation of Toxicogenomic Technologies, National Research Council

Washington, DC:National Academies Press, 2007. 98 pp. ISBN: 0-309-10413-0, $18

Veterinary Toxicology: Basic and Clinical Principles

Ramesh C. Gupta, ed.

Burlington, MA:Elsevier, 2007. 1,224 pp. ISBN: 0-12-370467-7, $99.95

What We Know About Climate Change

Kelly Emanuel

Cambridge, MA:MIT Press, 2007. 96 pp. ISBN: 0-262-05089-7, $14.95

